# Facial radiance influences facial attractiveness and affective impressions of faces

**DOI:** 10.1111/ics.12673

**Published:** 2020-12-26

**Authors:** Hanako Ikeda, Yuriko Saheki, Yuichi Sakano, Atsushi Wada, Hiroshi Ando, Keiko Tagai

**Affiliations:** ^1^ Shiseido Global Innovation Center Yokohama Kanagawa Japan; ^2^ Center for Information and Neural Networks (CiNet) National Institute of Information and Communications Technology Osaka University Suita Osaka Japan; ^3^ Graduate School of Frontier Biosciences Osaka University Suita Osaka Japan; ^4^ Center for Information and Neural Networks (CiNet) National Institute of Information and Communications Technology Osaka University Kyoto Japan

**Keywords:** claim substantiation, colour cosmetics, face, gloss, skin physiology, structure

## Abstract

**Objective:**

Facial attractiveness has been reported to be influenced by visual features such as facial shape and the colour and texture of the skin. However, no empirical studies have examined the effects of facial skin radiance on facial attractiveness. The present study investigated whether types of skin reflection (i.e. radiant, oily and shiny, and matte) and the position of the reflection on the face influence facial attractiveness and other affective impressions.

**Methods:**

A total of 160 female participants (1) estimated the ages and (2) evaluated attractiveness and other impressions of unfamiliar female faces in a total of seven skin reflection conditions. These conditions incorporated three types of reflection (i.e. radiant, oily and shiny, and matte) and three positions of the reflection on the face (i.e. entire facial skin, only cheeks, and only T‐zone).

**Results:**

The facial images of radiance on entire faces were rated as appearing younger than the facial images of oily shine on entire faces and the matte faces. Attractiveness ratings and other positive impressions increased in the order of the matte (ranked lowest), the oily shine on entire face, and the radiance on entire face (ranked highest) conditions. The reflection position also influenced facial attractiveness: attractiveness ratings and other positive impressions were higher in the radiance on entire face condition than in the radiant cheeks and the radiant T‐zone conditions. Interestingly, the radiant cheek faces were rated more radiant and healthier but less feminine and less bright than the radiant T‐zone faces.

**Conclusion:**

These results suggest that facial radiance enhances facial attractiveness and conveys a wide variety of positive impressions on the observer. The magnitude of the effects of cheek radiance and T‐zone radiance differs across various affective impressions. Nevertheless, the results demonstrate that cheek and the T‐zone radiance both contribute to higher attractiveness and other positive impressions of the radiance on entire faces. We believe that our findings can contribute as a guide to the enhancement of positive facial impressions by means of skin radiance, thereby leading to a better understanding of the value of skincare and base makeup.

## Graphical abstract

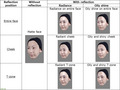

## Résumé

**Objectif:**

Selon certaines études, l’attractivité du visage viendrait de caractéristiques visuelles telles que la forme du visage ainsi que la couleur et la texture de la peau. Cependant, aucune étude empirique n’a examiné les effets de l’éclat de la peau sur l’attractivité du visage. La présente étude s’est intéressée aux divers types de reflets de la peau (par exemple une peau lumineuse, une peau grasse et luisante ou une peau mate) et à leur position sur le visage, et a cherché à savoir s’ils influençaient l’attractivité du visage et d’autres impressions d’ordre affectif.

**Méthodes:**

Au total, 160 participantes 1) ont estimé l’âge et 2) ont évalué l’attractivité et d’autres impressions de visages féminins inconnus sur sept types de reflets de la peau au total. Ces états comprenaient trois types de reflets (lumineux, gras et mat) et trois positions des reflets sur le visage (toute la peau du visage, joues uniquement et partie yeux‐nez [« zone T »] uniquement).

**Résultats:**

Les photos de visages caractérisées par un éclat sur tout le visage ont été considérées comme étant plus jeunes que celles dont la peau était grasse‐luisante sur l’ensemble du visage et que les visages à peau mate. Les notes attribuées pour l’attractivité et les autres impressions positives augmentent dans l’ordre suivant : peau mate (note la plus basse), peau grasse‐luisante sur tout le visage et luminosité sur tout le visage (note la plus haute). La position du reflet a elle aussi joué un rôle sur les notes attribuées à l’attractivité des visages, et les autres impressions positives ont obtenu des scores plus élevés pour une luminosité de tout le visage que pour des joues lumineuses et une zone T lumineuse. Il est intéressant de noter que les visages aux joues lumineuses ont été jugés plus lumineux et plus sains, mais moins féminins et moins éclatants que les visages présentant une zone T lumineuse.

**Conclusion:**

Ces résultats suggèrent que l’éclat du visage améliore l’attractivité du visage et communique toutes sortes d’impressions positives à l’observateur. L’ampleur des effets de l’éclat sur les joues et sur la zone T diffère selon les différentes impressions d’ordre affectif. Néanmoins, les résultats démontrent que la luminosité des joues et de la zone T contribue à rendre le visage plus séduisant ainsi qu’à d’autres impressions positives suscitées par la luminosité de l’ensemble du visage. Nous sommes convaincus que nos résultats peuvent servir à guider la façon de renforcer l’impression positive produite par un visage en faisant ressortir la luminosité de la peau, et amener ainsi à mieux comprendre la valeur des soins de la peau et des bases de maquillage.

## Introduction

What visual features affect facial attractiveness and other impressions when we see other people's faces? It is well known that the shape, colour and texture of the face influence facial attractiveness [1, 2, 3, 4]. Baudouin and Tiberghien [5] claimed that the female faces are considered attractive when they are symmetrical, close to the average in shape and have certain other features (e.g. large eyes, thick lips and thin eyebrows). Many other studies also have reported that average faces are regarded as attractive [6]. Facial colour distribution [2] and evenness of skin texture [7] have also been reported to affect facial attractiveness. With regard to visual features that influence impressions other than attractiveness, facial redness has been reported to enhance the healthy appearance of a face [8, 9], whereas facial blemishes seem to convey the impressions of poor health and low competence [7].

In addition to the above‐mentioned visual features (i.e. shape, colour and texture), facial skin reflection could also influence affective impressions, including attractiveness, because according to consumer cosmetics magazines, for instance, consumers seem to have a tendency to use skincare and base makeup products to enhance their facial radiance, which is a particular type of reflection [10, 11]. Such a preference for radiant skin could indicate a substantial link between facial skin reflection and affective impressions of faces, including attractiveness.

However, most previous studies on facial skin radiance focused solely on quantifying it based on physical quantities, rather than examining the effects of skin radiance on facial attractiveness. This is because skin radiance is a psychologically defined quantity, despite the fact that it is a type of optical skin reflection (or the psychologically determined category of this reflection). For instance, Baret and colleagues [12] proposed a mathematical model based on instrumental data to quantify the global radiance phenomenon. However, the model did not totally explain the global perception of skin radiance. Musnier and colleagues developed a methodology to evaluate complexion radiance based on trained assessors' ratings of skin colouring, luminosity, brightness and transparency [13]. They also applied this methodology to a cosmetic product to see whether it improved complexion radiance. To evaluate complexion radiance objectively, Petitjean and colleagues developed and evaluated an optical device that measured three radiance‐related indexes [14]. They found that one measured index was not repeatable and recommended that the same trained investigator always perform the measurements. Fujii and colleagues reported that high and low spatial frequency bands of specular reflection components of facial images represent texture (e.g. pores and wrinkles) and facial features (e.g. nose and cheekbones), respectively. They also reported that faces with high and low luminance contrasts in this high‐frequency band could be classified into oily and shiny, and radiant faces, respectively [15]. Matsubara et al. [16] reported that facial skin radiance requires a balance between the mean and the standard deviation of luminance across the face that arises from the skin’s subsurface and surface reflections, respectively. Similarly, Masuda et al. [17] reported that facial skin regarded as radiant showed both specularly and diffusely reflected light from the face, and the measurement of these reflected lights is crucial for the measurement of skin radiance. Haeri et al. [18] proposed a new approach called fractal analysis to characterize facial skin radiance and reported the advantage of this new analysis over the one based on classical shine when colour evenness is an important cue to radiance.

In sum, most previous studies on facial radiance tried to quantify it based on physical quantities, rather than examine effects of skin radiance on facial attractiveness and other affective impressions. The main purpose of the present study was to clarify the effects of facial skin radiance on facial attractiveness, rather than to explore the definition of skin radiance based on physical quantities.

Although facial radiance can create a good impression [10], another type of reflection, so called ‘oily and shiny gloss’, may make a bad one [19, 20, 21]. Torizuka and co‐workers [20] have shown that females are conscious of shiny skin when their makeup deteriorates. Fujii and colleagues reported that increasing and decreasing the luminance contrast in the high‐frequency band of facial images resulted in increasing and decreasing negative impression of the face, respectively. Combined with their finding based on the other experiment mentioned above, where faces with a high luminance contrast in this high‐frequency band were classified into oily and shiny faces, their results could indicate that the oily and shiny condition increases the negative impression of the face [15]. Arce‐Lopera and co‐workers (2012) reported that eliminating the specular component from cropped photographs of enlarged facial skin elicited a younger impression, presumably because wrinkles became practically invisible. This type of specular image component may constitute oily and shiny gloss [19]. However, using cropped photos of partial facial skin could miss the potential effects of the position of skin reflection on the face. It has been reported that cheekbone prominence and height are positively correlated with attractiveness [5]. Those chiselled facial features can change the position of reflection on the face. Therefore, we also examined the effects of the position of skin reflection on facial attractiveness and other affective impressions.

Another type of skin reflection that has a growing awareness among cosmetic consumers is the property of matte. Matte skin makes little impression of radiance or the oily and shiny condition because of lack of specular reflection [16].

The purpose of the present study was to elucidate whether the types of facial skin reflection (i.e. radiant, oily and shiny, and matte) and the position of the reflection on the face influence facial attractiveness and other affective impressions. To this end, we conducted two psychological experiments with 160 female participants each. First, we examined the effects of skin reflection types and positions on estimated age. Second, we comprehensively investigated those effects on facial attractiveness and on nine other affective impressions, including the desire to compliment, looking happy, likableness, looking tired, looking feminine, looking healthy, skin radiance, oily shine and skin brightness.

## Methods

### Participants

A total of 160 adult females between the ages of 30 and 49 years (M = 39.65, SD = 5.86) were recruited for the study. All participants were paying more than 1,000 yen per month on skincare products and used them for more than five days a week. They all lived in Japan, with Japanese as their native language. All were healthy, had no history of neurological disorders and had normal or corrected‐to‐normal vision. All gave written informed consent before each experiment. The experimental procedures were approved by the Ethical Committee of the Shiseido Global Innovation Center.

### Apparatus

All visual images and questionnaires were presented to each participant on a 24.1‐in (1920 × 1200 pixels) liquid‐crystal display. Images of faces were presented at a resolution of 719 × 1078 pixels on a grey (RGB (128, 128, 128)) background. Ten displays and ten computers were used to run the experiments for ten participants simultaneously. The displays were colour calibrated with an X‐rite i1 Pro (X‐Rite Inc.) to ensure the same colour conditions for all displays. We adjusted the maximum luminance and gamma to 160 cd m^−2^ and 2.2, respectively. The order of presentation of the photo images was controlled with a computer program.

### Facial stimuli

**Figure 1:**
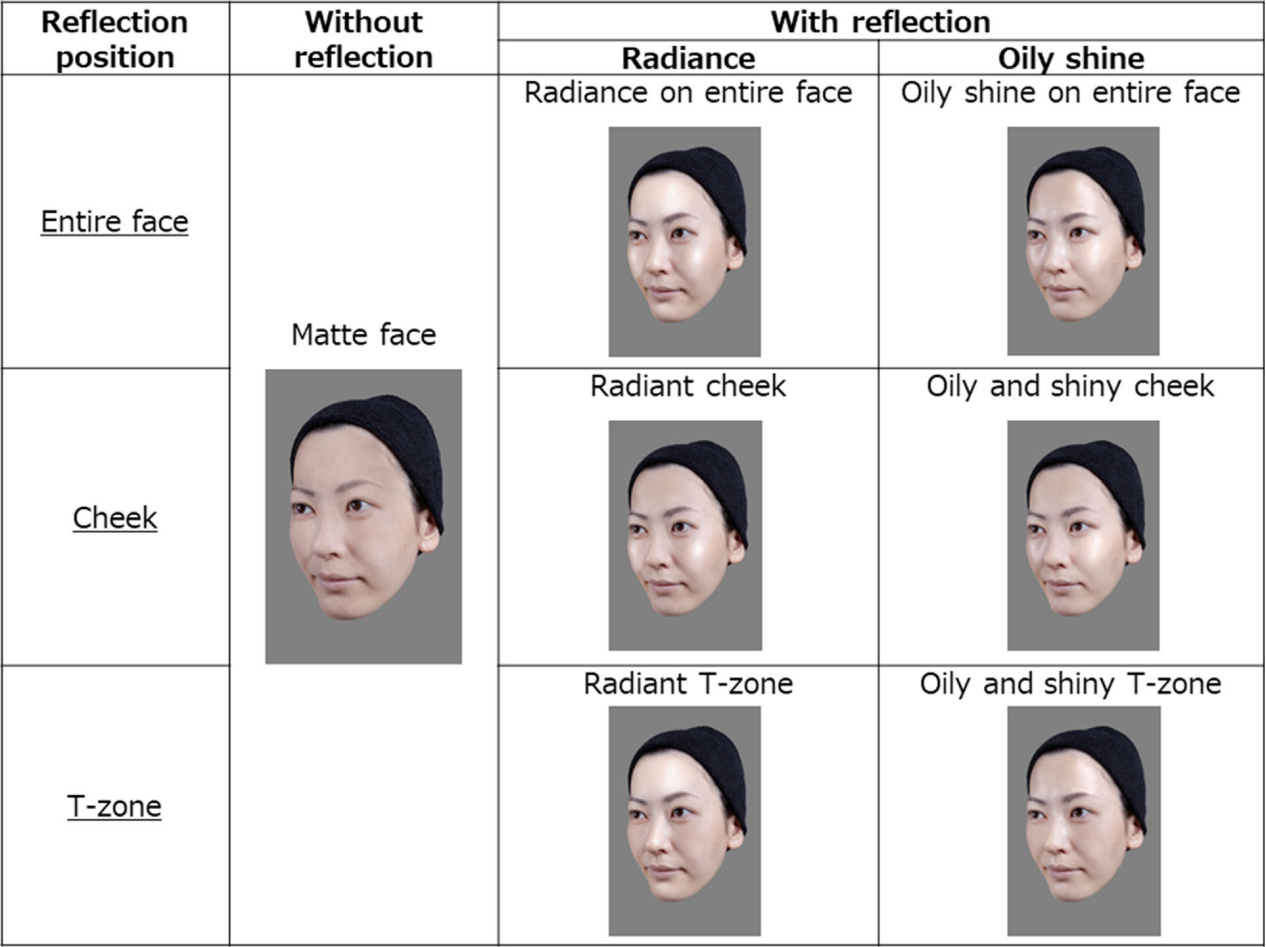
Examples of stimuli used in the experiment. The subject gave informed consent to publish images of her face

Images used in this experiment featured ten adult female models between the ages of 30 and 44 years (M = 35.90, SD = 4.44). They all lived in Japan, and all gave written informed consent before each photography session. The procedures were approved by the ethical committee of the Shiseido Global Innovation Center. Figure 1 shows three conditions of facial gloss (radiance, oily shine and matte) and three positions of gloss (T‐area, cheek area, entire area). A ‘matte face’ is a face without gloss. The T‐area refers to the forehead and bridge of the nose. In our experiment, these seven types of faces were used as facial stimuli. To avoid subtle differences in facial expressions and other features among faces with different types of gloss, we created a base face for each female model. The seven types of faces shown in Figure 1 were created by retouching the base face.

The process of preparing photo stimuli was composed of two phases. In the first phase, which was the photographing phase, the faces of the female models were photographed in several poses with different cosmetics applied to them under different lighting conditions. All sty

les of makeup, excluding skincare, were manipulated by a professional makeup artist. In the second phase, which was the retouching phase, the photographs were retouched by a computer graphics technique. The images of all ten female models were subjected to the same process. In the photography phase, we collected image materials for a base face image as well as many types of skin reflection by changing the lighting and applying different cosmetics. In the retouching phase, we first created a base face image, which was not used in the experiment but used for preparing photographs presented in the experiment. All types of face used in the experiment were created by adding and/or removing reflections from the base face image.

#### Photography phase

All female models rotated their bodies to the horizontal‐right direction, 45 degrees from the frontal angle, for the gloss to appear on the skin in the photographs. Thus, the left side of the face was captured by the camera. To control for the same facial angle for all participants, a chin rest was used. All female models sat, whereas the pictures were taken by a digital single‐lens reflex Canon 5D camera (Canon Inc.) with 50 mm lens (Tamron Co., Ltd).

To express the different types of gloss on a face, we prepared several lighting conditions and cosmetics, and combined the conditions. The results of previous studies [15, 17, 22] were confirmed here as references to choose the lighting conditions and cosmetics to apply to the face. In previous studies, observers had reported their different impressions, such as radiant and oily shine, depending on a combination of the heights of specular reflection light and diffuse reflection light generated on the skin of the face. A study by Masuda et al. showed that when specular reflection light was abundant, the impression of an oily and shiny skin was more prominent, and when diffuse reflection light was abundant, the impression of transparent skin was evoked [17]. In addition, skin containing both types of reflections at high degrees appeared radiant and appeared dull when both were low [17].

Diffuse reflection light is multiple reflection where the source is unclear. Skin with a higher degree of diffuse reflection showed characteristics of more moisture and fine texture [23]. Unlike specular reflection, it did not have a small amount of surface information on irregular skin [24]. The specular reflection light turned into strong reflected light. Texture roughness has been reported as characteristic of skin with higher specular reflection light and lower diffuse reflection light [17]. In addition, specular reflection light images showed the texture of the skin using a small surface shape and by the sebum distributed on the skin surface [15].

To artificially express the characteristics of diffuse reflection of light on the skin, it was necessary to produce reflected light that did not prominently convey the feeling of skin roughness. For this purpose, we used diffused illumination for photography and applied cosmetic products that could smooth facial skin. To create a radiant image of the face, we manipulated the intensity of illumination of directional reflected light to express specular reflection light, in addition to diffuse reflection light. In contrast, to generate strong specular reflection characteristics, light that was not diffused was used. A previous study on the relation between impressions of facial skin and skin biology [25, 26], and deterioration in makeup [20] suggests a positive correlation between the impression of facial shine (oily, shiny gloss) and the amount of facial sebum. Therefore, cosmetics containing large amounts of oil in the base material were applied to the facial skin to generate specular reflection characteristics for oily and shiny faces. Photography was carried out by combining the illumination conditions below with the cosmetic patterns applied to the face.

All female models were photographed with neutral facial expressions and smiling faces. When the female models were smiling, there was particularly strong specular reflection because of increased convexity of the cheeks.

##### Illumination conditions during photography

**Figure 2:**
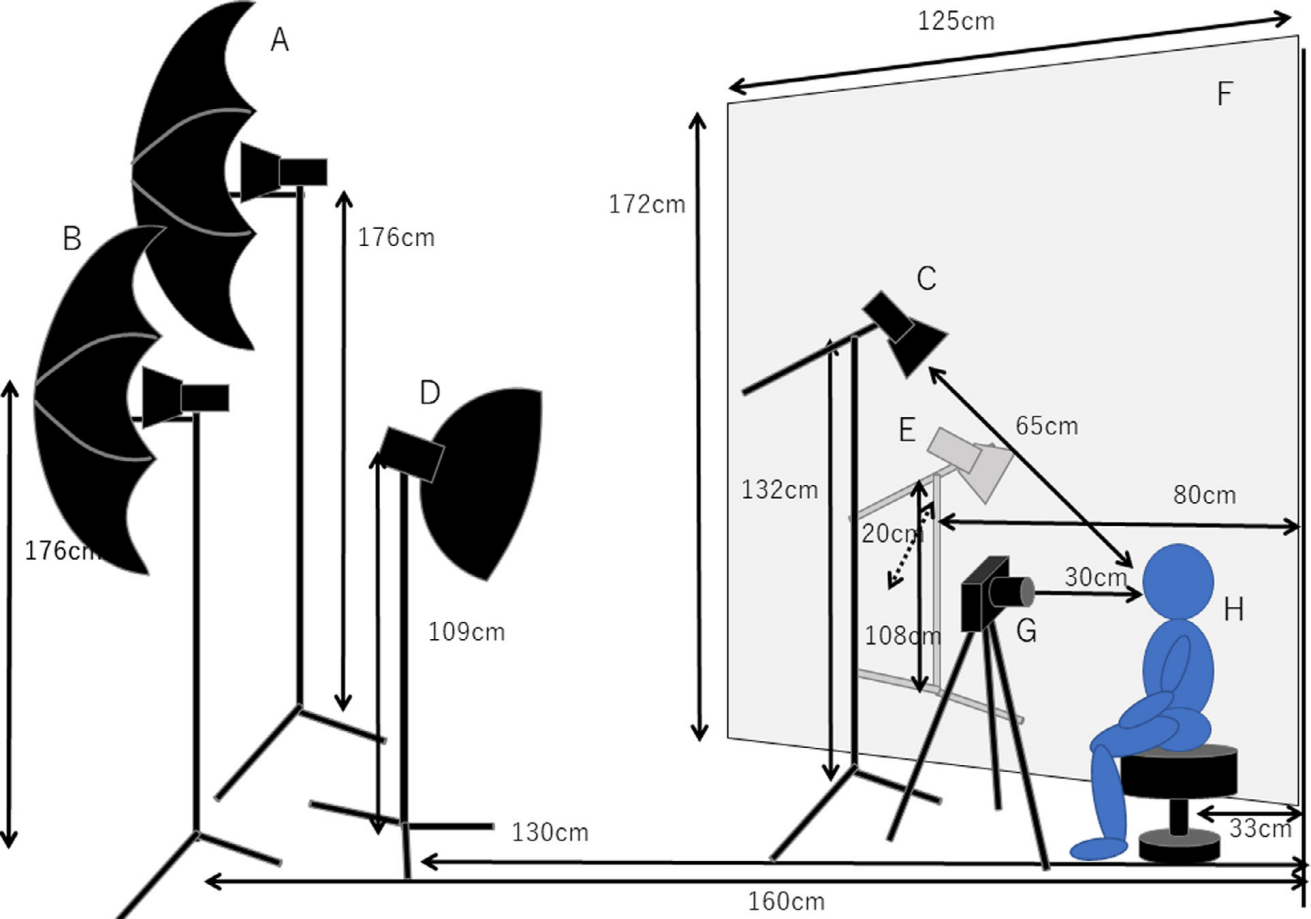
This figure shows the arrangement of the photo shooting setting. A and B were strobe lights fitted with umbrella‐shaped reflectors. C was a strobe light. A, B and C were used in the condition of non‐diffusion lighting. D was a strobe light equipped with a diffuser. E was a strobe light and put behind a cloth diffuser. F was a cloth diffuser. D and E were used in the condition of diffused lighting. G was a camera and H was a model

All female models were photographed under non‐diffuse illumination conditions (intense and non‐diffuse illumination) and under diffused illumination (light diffusing illumination). Under non‐diffusion lighting conditions, in addition to a strobe light (COMET strobe light and strobe head), two strobe lights fitted with umbrella‐shaped reflectors were used. All three lamps were used at an output of 200 W. In the diffused lighting condition, one side‐light from the other side of a diffuser cloth and a strobe light equipped with a diffuser were used at an output of 112 W. Figure 2 shows the arrangement of illuminations, camera and model.

##### Cosmetic products applied to female models’ faces

All female models washed their faces before being photographed, and then applied 2 mL of lotion (ELIXIR SUPERIEUR LIFTING MOISTURE LOTION W II) and 1.4 mL of emulsion (ELIXIR SUPERIEUR LIFTING MOISTURE EMULSION W II) to their faces to avoid dryness. They then rested for about ten minutes until the redness of the skin decreased. All styles of makeup were manipulated by a professional makeup artist without skincare. Following this, the artist drew eyebrows on the female models to avoid the influence of faces without eyebrows on facial impression. Actually, some models changed the shape of their eyebrow by removing some parts of their natural eyebrow shape. They were then photographed in three patterns of applied cosmetics: applying only solid foundation (cle de peau BEAUTE RADIANT STICK FOUNDATION) to the skin, applying cosmetic 0.2 mL of cosmetic oil (Carita14) to prevent dry skin over the foundation and applying cosmetic moisturizing gel (ELIXIR SUPERIEUR SLEEPING GEL PACK W) without white particles only on the cheeks, in addition to about 0.2 mL of cosmetic oil (Carita14) above the foundation.

In a trial photoshoot, foundation applied to the skin was smoothed and impressions of the smooth texture of the skin were observed. The photographed images of the face with only foundation applied to it were used as the material for diffuse reflection light to generate a radiant face and the base face. The trial photoshoot confirmed that faces coated with cosmetic oil and gel yielded reflection images showing oily and shiny skin, and irregularities on the skin surface. Cosmetic oil and gel were thus used to represent specular reflection light for oily and shiny faces.

#### Retouching images phase

Digital images of the face were retouched by computer graphics (CG) software (Adobe Photoshop CC2017). The gamma value of the display Color Edge CG243W (Eizo Corporation) used for retouching was 2.2. We created a base face for each female model and glossy images under the three conditions by transplanting various highlighted materials from photos of the same female model to the base face. The highlighted materials were transplanted on the convex part of the face, including the forehead, ridge of the nose, cheek and chin. The ranges of the transplanted highlights were specified to naturally fit the shape of the face of each female model. In addition to one person being in charge of image retouching, five people, the three authors and two members of the staff of Shiseido Company Limited, confirmed whether the range, shape and strength of the highlights were appropriate.

##### Method to create base faces

A base face was created by removing strong highlights in the convex parts of images of faces with a neutral expression and only foundation applied under diffusion illumination light conditions. We transplanted the image of the skin of the non‐highlighted portion onto the highlighted one. However, the natural highlight was left unchanged. Skin eruptions, spots and scratches were also removed.

##### Method to create radiant faces

Images of radiant faces were created by copying glossy skin parts of a female model’s face in an image and applying it to the base face of same model’s face by retouching using the CG software. Glossy parts of the skin in the images, used as materials for creating radiant faces, were generated by combining different types of reflected lights. One had the characteristics of diffuse reflection light and another those of strong specular reflection light on facial skin. Gloss with the characteristic of diffuse reflected light was cut out from the foundation applied to the skin of the face under diffuse illumination and transplanted to the base face. Furthermore, specular reflection occurring on skin with foundation applied to it (specifically in images of smiling faces) under strong non‐diffused lighting was cut out and transplanted to the base face.

##### Method to create oily and shiny faces

Images of oily and shiny faces were created by applying oily and shiny glossy skin parts of a female model’s face to her base face. Glossy skin parts as materials for creating oily and shiny faces were generated from skin that exhibited features of specular reflection, was oil‐rich, and had remarkable irregularities. Images of oily and shiny gloss materials were cut out from facial images featuring smiling faces, with foundation and oil or foundation, oil, and gel applied, that had been shot under non‐diffuse illumination, and these materials were transplanted to the base face.

##### Method to create matte faces

We removed all highlights from the base face, including lustre.

### Tasks and procedures

Each participant estimated the age of a female model through photographs in Task 1 and evaluated various impressions evoked by the photographs in Task 2, where the Scheffe method for paired comparison tests (the modified Nakaya method) [27, 28] was used.

All participants performed both tasks and answered each question on a computer. Each participant sat one metre from the computer display. The 160 participants were divided into 10 groups of 16 each. They observed all seven types of faces (Figure 1) of one of the ten female models. Therefore, each participant saw photographs of only one model’s face throughout the experiment. The age distribution was roughly equal across all groups. The participants were instructed to answer with their intuitive impressions of images of faces evoked by the skin.

#### Task 1–Estimating age

An image of a face in seven image conditions was presented on a computer display for five seconds. After the image disappeared, the participants were asked to estimate the age of the person in the image from zero to 80 years. There was no time limit for the answer. The participants thus performed seven trials in the task because each participant observed all seven images of all seven conditions of the same facial model. These images were presented in a randomized order to each participant.

#### Task 2–Evaluating impression

**Figure 3:**
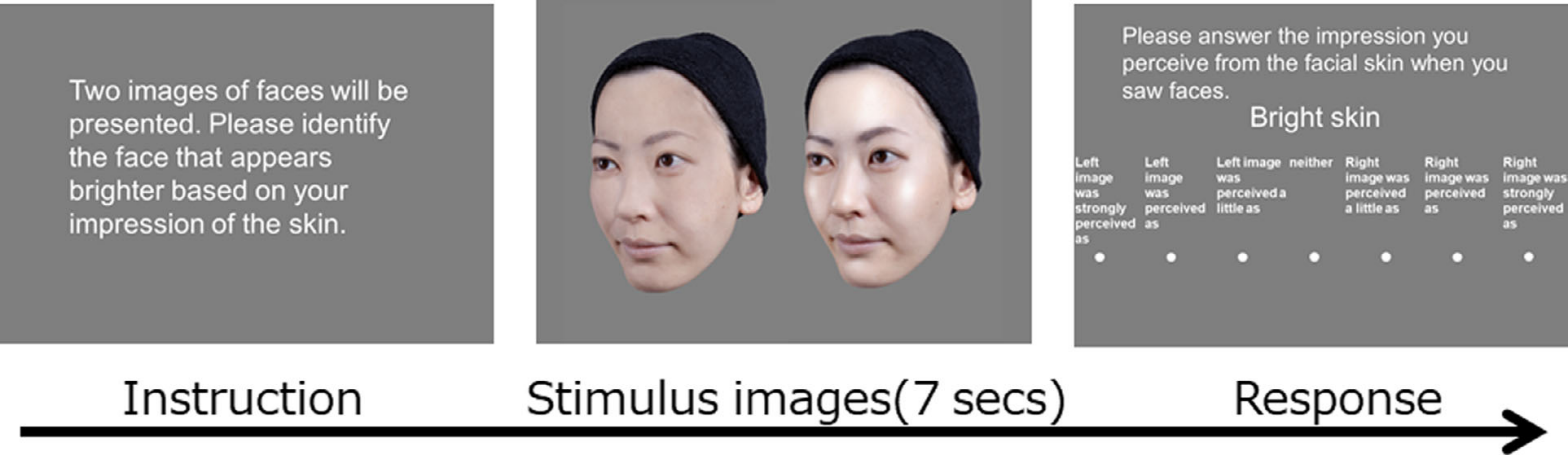
An example of the procedure of Task 2. The instructions were in Japanese. Participants had to evaluate degree of impression expressed by a word on the centre of display after they saw two facial images

In this task, the participants were asked to evaluate the degrees of the following ten types of impressions when they observed images of faces: ‘Attractive’, ‘Want to compliment’, ‘Happy’, ‘Likable’, ‘Tired’, ‘Feminine’, ‘Healthy’, ‘Radiant skin’, ‘Oily and shiny skin’ and ‘Bright skin’. We categorized ‘Attractive’, ‘Want to compliment’, ‘Happy’, ‘Likable’, ‘Tired’, ‘Feminine’ and ‘Healthy’ as abstract words, and ‘radiant skin’, ‘Oily and shiny skin’ and ‘Bright skin’ as words representing sensory descriptors. In each trial, two faces from the seven image conditions of a female model were presented simultaneously on the computer display for seven seconds. Once the faces disappeared, the participants were asked to determine which face evoked a stronger impression in terms of the specified type of impression, and to score this on a seven‐grade scale (‘Left image was strongly perceived as’ – ‘Left image was perceived as’ – ‘Left image was perceived a little as’ – ‘Neither’ – ‘Right image was perceived a little as’ – ‘Right image was perceived as’ – ‘Right image was strongly perceived as’) (Figure 3). The task procedure followed the Scheffe method for a paired comparison test (the modified Nakaya method) [27, 28]. In total, 21 pairs were created among all seven images of the same facial identity, representing all possible pair‐wise combinations. The participants answered ten questions for each image combination made from one same photograph model. Therefore, they performed 210 trials in total. For each participant, the questionnaire was switched once she had completed to answer for all 21 pairs. The order of questions was randomized for each participant, as was the order of presentation of the paired images in each question.

## Results

### Task 1

**Figure 4:**
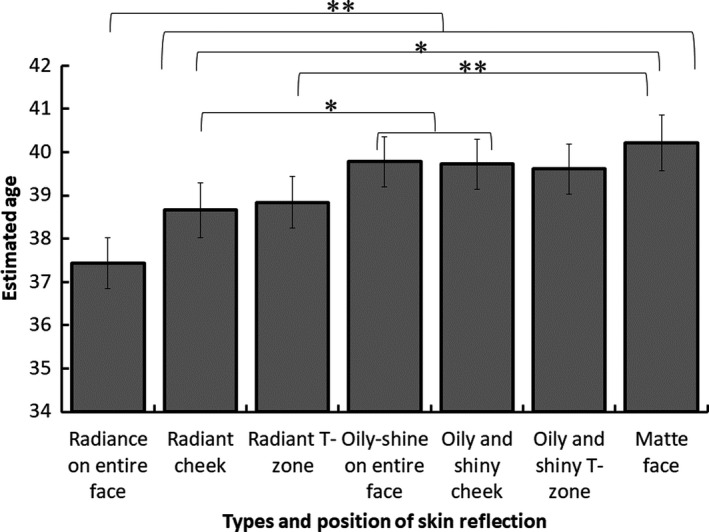
Estimated age of model in each face image. Error bars indicate ± 1 SEM across participants. *Note*: * *P* < 0.05, ** *P* < 0.01

Figure 4 shows the estimated age of the female model in each image condition averaged across all participants. One‐way repeated measures of ANOVA indicated a significant main effect of the image conditions (*F*(6,954) = 18.59, *P* < 0.001). Bonferroni post‐hoc tests revealed that the faces with radiance of the entire face were estimated as the youngest in all image conditions (*ps* < 0.005). Faces with radiant cheeks were evaluated to be significantly younger than oily and shine on entire faces, faces with oily and shiny cheeks, and matte faces (*ps* < 0.03). However, there were no significant differences between assessments of faces with radiant cheeks and those with radiant T‐zones, and with oily and shiny T‐zones (*ps* > 0.13). Faces with radiant T‐zones were evaluated to be significantly younger than matte faces (*p* < 0.001) but did not show significant differences from assessment of oily shine on entire faces, faces with oily and shiny cheeks, and those with oily and shiny T‐zones (*ps* > 0.08). There was no significant difference among evaluations of oily shine on entire faces, faces with oily and shiny cheeks, those with oily and shiny T‐zones, and matte faces (*ps = *1).

### Task 2

**Figure 5:**
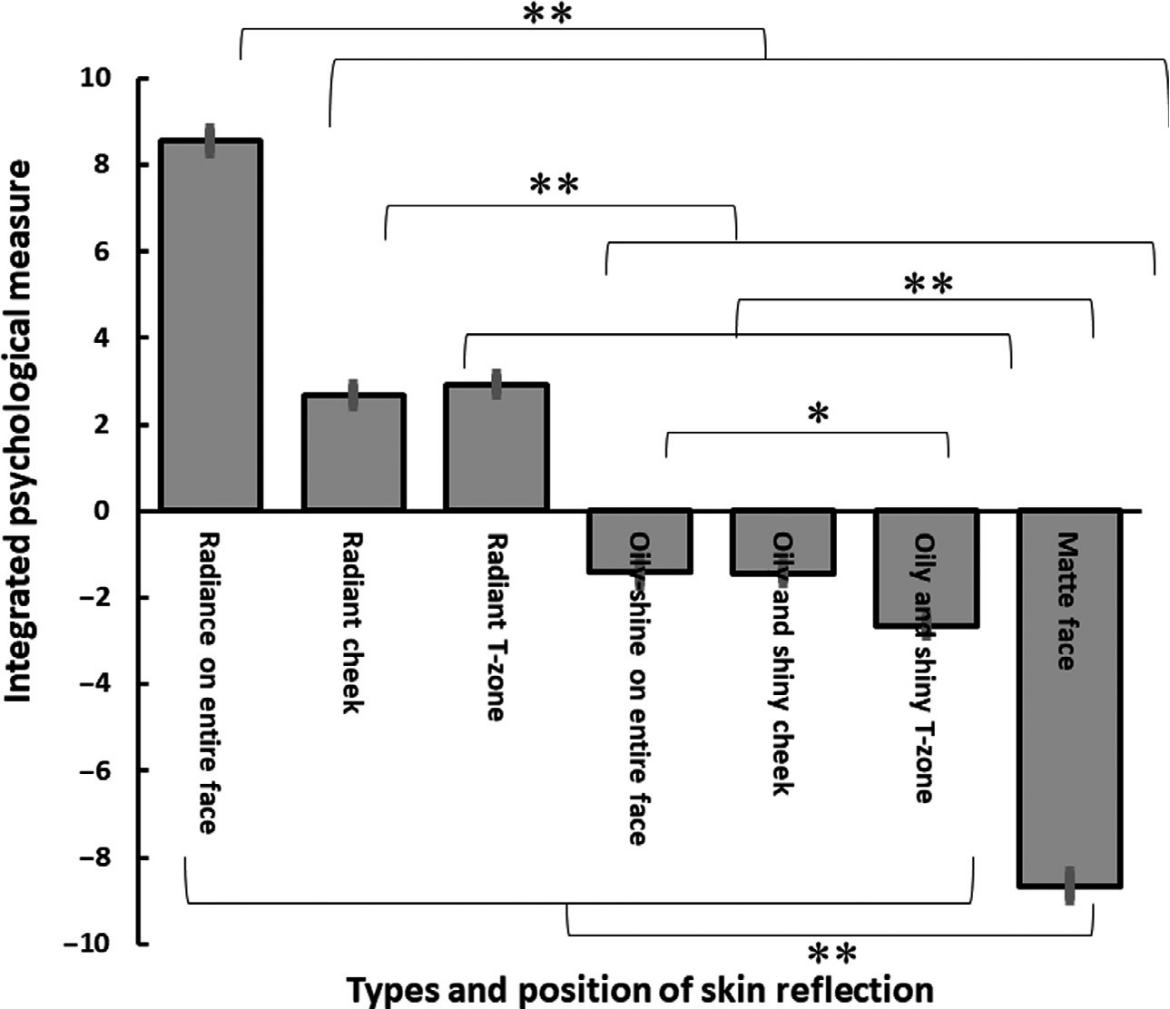
Results of Task 2. Sums of preference values of seven abstract words (‘Attractive’, ‘Want to compliment’, ‘Happy’, ‘Likable’, ‘Tired’, ‘Feminine’ and ‘Healthy’) are showed in each face image. Error bars indicate ± 1 SEM across participants. *Note*: * *P* < 0.05, ** *P *< 0.01

In order to overview the degree of positive (or negative) impressions for the seven conditions of facial stimuli, we have devised an integrated positive‐negative measure that summed up preference values of seven abstract words (‘Attractive’, ‘Want to compliment’, ‘Happy’, ‘Likable’, ‘Tired’, ‘Feminine’ and ‘Healthy’). We reversed the sign of the value for the impression ‘tired’, as it represents a negative impression. Figure 5 shows averaged result of them among all participants. One‐way repeated measures of ANOVA indicated a significant main effect of the image conditions (*F*(6,954) = 326.6, *P* < 0.001). Bonferroni post‐hoc tests showed no significant differences either between the faces with radiant cheeks and radiant T‐zones (*P* = 1), between the faces with oily and shiny cheeks and oily and shiny T‐zones, or between the faces with oily and shiny cheeks and oily shine on entire faces (*ps* > 0.05). All pairs other than the above showed significant differences (*ps* < 0.05). These results suggest that the faces with radiance of the entire face create the most positive impression in all image conditions. The radiant cheeks and T‐zones were rated more positive than all the faces with oily shine and the matte face. The oily and shiny T‐zone was rated more negative than the oily shine on the entire face. The matte face created the most negative impression. More detailed results of each questionnaire were shown below.

**Figure 6:**
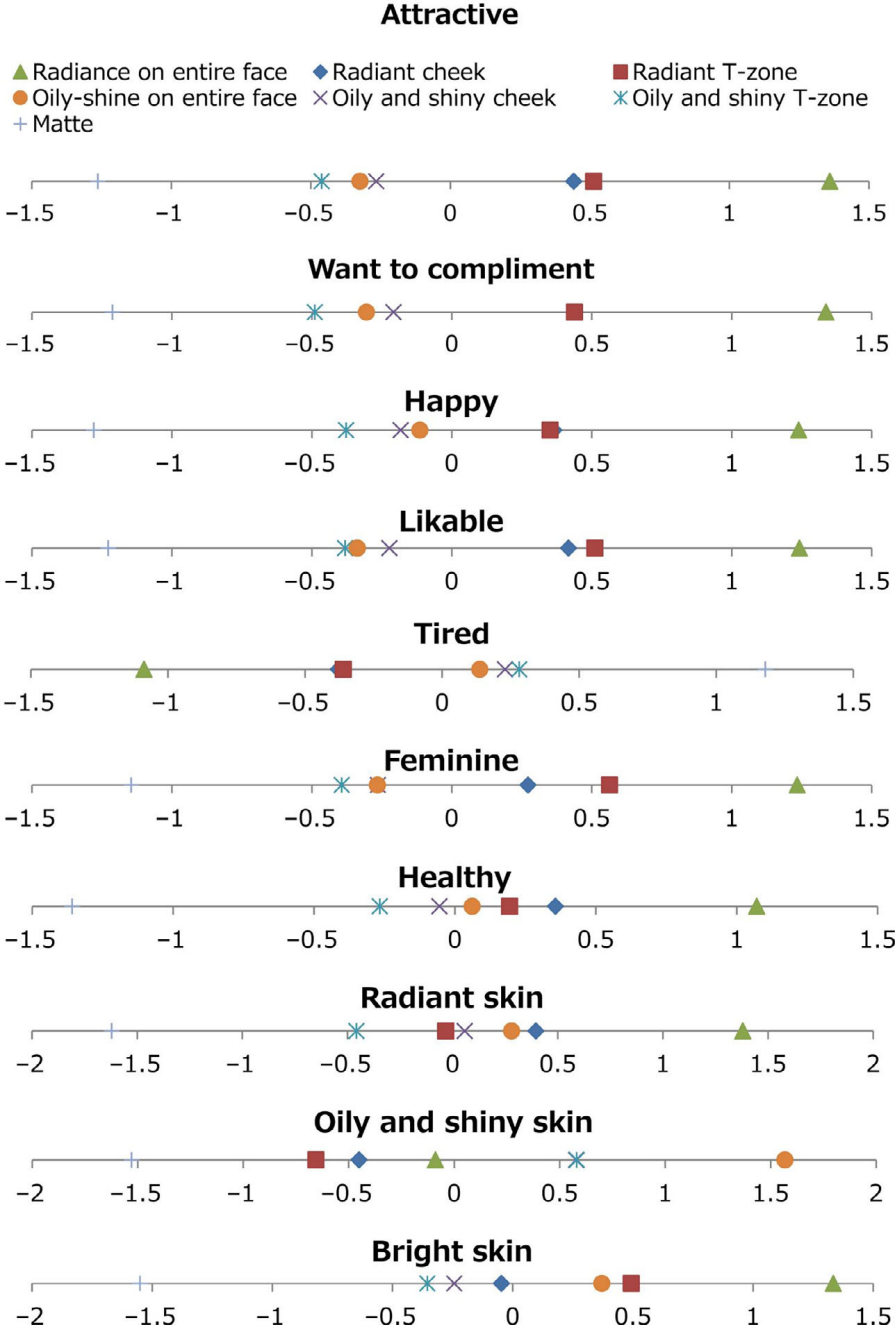
Detailed results of Task 2. Each number line shows psychological measures for all image conditions with respect to each impression

Figure 6 shows the detailed results of Task 2. Each point on each horizontal bar indicates the result of each image condition on each question. Larger values imply stronger impressions conveyed by images of faces. On all impression‐related questions, the one‐way repeated measures ANOVA showed a significant effect of image condition (*P* < 0.001). This suggests significant differences among the image conditions. The yardstick values were calculated, and significant differences were found between certain conditions. Table 1 shows differences among conditions of the images.

**Table 1:** Differences in scores of paired comparison on Task 2. There were significant results at a 5% yardstick interval (*) and a 1% yardstick interval (**). (a)–(j) indicated each result of all questionnaires

Attractive		Radiance on entire face	Radiant cheek	Radiant T‐zone	Oily – shine on entire face	Oily and shiny cheek	Oily and shiny T‐zone	Matte face
Image condition	Score	Score difference	Score difference	Score difference	Score difference	Score difference	Score difference	Score difference
(a)								
Radiance on entire face	1.36		0.92**	0.85**	1.68**	1.63**	1.82**	2.62**
Radiant cheek	0.44			−0.07	0.77**	0.71**	0.90**	1.71**
Radiant T‐zone	0.51				0.84**	0.78**	0.98**	1.78**
Oily – shine on entire face	−0.32					−0.06	0.14**	0.94**
Oily and shiny cheek	−0.27						0.20**	1.00**
Oily and shiny T‐zone	−0.46							0.80**
Matte face	−1.26							

**Want to compliment**		Radiance on entire face	Radiant cheek	Radiant T‐zone	Oily – shine on entire face	Oily and shiny cheek	Oily and shiny T‐zone	Matte face
Image condition	Score	Score difference	Score difference	Score difference	Score difference	Score difference	Score difference	Score difference
(b)								
Radiance on entire face	1.34		0.90**	0.90**	1.64**	1.54**	1.83**	2.55**
Radiant cheek	0.44			0.00	0.75**	0.65**	0.93**	1.65**
Radiant T‐zone	0.44				0.74**	0.65**	0.93**	1.65**
Oily – shine on entire face	−0.31					−0.10	0.18**	0.91**
Oily and shiny cheek	−0.21						0.28**	1.00**
Oily and shiny T‐zone	−0.49							0.72**
Matte face	−1.21							

**Happy**		Radiance on entire face	Radiant cheek	Radiant T‐zone	Oily – shine on entire face	Oily and shiny cheek	Oily and shiny T‐zone	Matte face
Image condition	Score	Score difference	Score difference	Score difference	Score difference	Score difference	Score difference	Score difference
(c)								
Radiance on entire face	1.24		0.88**	0.89**	1.35**	1.42**	1.62**	2.52**
Radiant cheek	0.36			0.01	0.48**	0.55**	0.74**	1.64**
Radiant T‐zone	0.35				0.47**	0.53**	0.73**	1.63**
Oily – shine on entire face	−0.11					0.07	0.26**	1.17**
Oily and shiny cheek	−0.18						0.19**	1.10**
Oily and shiny T‐zone	−0.38							0.90**
Matte face	−1.28							

**Likeable**		Radiance on entire face	Radiant cheek	Radiant T‐zone	Oily – shine on entire face	Oily and shiny cheek	Oily and shiny T‐zone	Matte face
Image condition	Score	Score difference	Score difference	Score difference	Score difference	Score difference	Score difference	Score difference
(d)								
Radiance on entire face	1.24		0.83**	0.73**	1.58**	1.47**	1.62**	2.47**
Radiant cheek	0.42			‐0.09	0.75**	0.64**	0.80**	1.64**
Radiant T‐zone	0.51				0.85**	0.73**	0.89**	1.74**
Oily – shine on entire face	−0.34					−0.11*	0.04	0.89**
Oily and shiny cheek	−0.22						0.16**	1.00**
Oily and shiny T‐zone	−0.38							0.85**
Matte face	−1.23							

**Tired**		Radiance on entire face	Radiant cheek	Radiant T‐zone	Oily – shine on entire face	Oily and shiny cheek	Oily and shiny T‐zone	Matte face
Image condition	Score	Score difference	Score difference	Score difference	Score difference	Score difference	Score difference	Score difference
(e)								
Radiance on entire face	−1.09		−0.71**	−0.73**	−1.23**	−1.32**	−1.37**	−2.27**
Radiant cheek	−0.38			−0.02	−0.52**	−0.61**	−0.66**	−1.56**
Radiant T‐zone	−0.36				−0.50**	−0.59**	−0.64**	−1.54**
Oily – shine on entire face	0.14					−0.09	−0.14**	−1.04**
Oily and shiny cheek	0.23						−0.05	−0.95**
Oily and shiny T‐zone	0.28							−0.90**
Matte face	1.18							

**Feminine**		Radiance on entire face	Radiant cheek	Radiant T‐zone	Oily – shine on entire face	Oily and shiny cheek	Oily and shiny T‐zone	Matte face
Image condition	Score	Score difference	Score difference	Score difference	Score difference	Score difference	Score difference	Score difference
(f)								
Radiance on entire face	1.23		0.96**	0.67**	1.50**	1.50**	1.63**	2.38**
Radiant cheek	0.27			−0.29**	0.54**	0.54**	0.67**	1.42**
Radiant T‐zone	0.56				0.83**	0.83**	0.96**	1.71**
Oily – shine on entire face	−0.27					0.00	0.1276*	0.88**
Oily and shiny cheek	−0.26						0.13**	0.88**
Oily and shiny T‐zone	−0.39							0.75**
Matte face	−1.15							

**Healthy**		Radiance on entire face	Radiant cheek	Radiant T‐zone	Oily – shine on entire face	Oily and shiny cheek	Oily and shiny T‐zone	Matte face
Image condition	Score	Score difference	Score difference	Score difference	Score difference	Score difference	Score difference	Score difference
(g)								
Radiance on entire face	1.07		0.71**	0.88**	1.01**	1.13**	1.34**	2.43**
Radiant cheek	0.36			0.16**	0.30**	0.41**	0.62**	1.72**
Radiant T‐zone	0.19				0.1331*	0.25**	0.46**	1.55**
Oily – shine on entire face	0.06					0.12	0.33**	1.42**
Oily and shiny cheek	−0.06						0.21**	1.30**
Oily and shiny T‐zone	−0.27							1.09**
Matte face	−1.36							

**Radiant skin**		Radiance on entire face	Radiant cheek	Radiant T‐zone	Oily – shine on entire face	Oily and shiny cheek	Oily and shiny T‐zone	Matte face
Image condition	Score	Score difference	Score difference	Score difference	Score difference	Score difference	Score difference	Score difference
(h)								
Radiance on entire face	1.38		0.98**	1.41**	1.10**	1.32**	1.84**	3.00**
Radiant cheek	0.40			0.43**	0.1151*	0.34**	0.85**	2.02**
Radiant T‐zone	−0.03				−0.31**	−0.09	0.43**	1.59**
Oily – shine on entire face	0.28					0.22**	0.74**	1.90**
Oily and shiny cheek	0.06						0.52**	1.68**
Oily and shiny T‐zone	−0.46							1.16**
Matte face	−1.62							

**Oily and shiny skin**		Radiance on entire face	Radiant cheek	Radiant T‐zone	Oily – shine on entire face	Oily and shiny cheek	Oily and shiny T‐zone	Matte face
Image condition	Score	Score difference	Score difference	Score difference	Score difference	Score difference	Score difference	Score difference
(i)								
Radiance on entire face	−0.09		0.36**	0.57**	−1.66**	−0.67**	−0.67**	1.44**
Radiant cheek	−0.45			0.20**	−2.02**	−1.03**	−1.03**	1.08**
Radiant T‐zone	−0.66				−2.22**	−1.23**	−1.24**	0.87**
Oily – shine on entire face	1.57					0.99**	0.99**	3.10**
Oily and shiny cheek	0.58						0.00	2.11**
Oily and shiny T‐zone	0.58							2.11**
Matte face	−1.53							

**Bright skin**		Radiance on entire face	Radiant cheek	Radiant T‐zone	Oily – shine on entire face	Oily and shiny cheek	Oily and shiny T‐zone	Matte face
Image condition	Score	Score difference	Score difference	Score difference	Score difference	Score difference	Score difference	Score difference
(j)								
Radiance on entire face	1.33		1.38**	0.84**	0.96**	1.58**	1.69**	2.88**
Radiant cheek	−0.05			−0.54**	−0.42**	0.20**	0.31**	1.50**
Radiant T‐zone	0.49				0.12	0.74**	0.85**	2.04**
Oily – shine on entire face	0.37					0.61**	0.73**	1.92**
Oily and shiny cheek	−0.24						0.11	1.31**
Oily and shiny T‐zone	−0.36							1.20**
Matte face	−1.55							

‘Attractive’, ‘Want to compliment’, ‘Happy’, ‘Likable’, ‘Feminine’ and ‘Healthy’ indicated similar tendencies. These impressions were felt most strongly in cases where the entire face, the cheeks and the T‐zone were radiant. Conversely, the three conditions featuring oily and shiny faces provided weaker impressions of ‘Attractive’, ‘Want to compliment’, ‘Happy’, ‘Likable’, ‘Feminine’ and ‘Healthy’, and the condition with the matte face evoked the weakest such impressions.

‘Attractive’, ‘Want to compliment’ and ‘Happy’ showed no significant differences between the conditions involving images of faces with radiant cheeks and T‐zones. There was no significant difference between the conditions of images with oily and shiny cheeks and those with oily shine on entire faces in the questions. Images of faces with oily and shiny T‐zones evoked significantly weaker impressions concerning ‘Want to compliment’, ‘Happy’ and ‘Attractive’ than other conditions involving oily and shiny faces (Table 1, Figure 6).

‘Likable’ exhibited no significant difference between the images of faces with radiant cheeks and T‐zones. There was no significant difference between images with oily and shiny T‐zones and oily shine on entire faces. Images of faces with oily and shiny cheeks evoked stronger impressions of ‘Likable’ than other oily and shiny faces (Table 1, Figure 6).

‘Feminine’ and ‘Healthy’ showed no significant differences between images with oily and shiny cheeks and oily shine on entire faces. Images with oily and shiny T‐zones evoked weaker impressions of ‘Feminine’ and ‘Healthy’ than other oily and shiny faces. ‘Feminine’ was felt significantly more strongly in images of faces with radiant T‐zones than those with radiant cheeks, and ‘Healthy’ was felt significantly more strongly in images with radiant cheeks than T‐zones (Table 1, Figure 6).

‘Tired’ was felt most strongly in images of matte faces, and in the three conditions featuring oily and shiny faces than the other conditions. Images of faces with radiant cheeks and T‐zones evoked weaker impressions, and images with radiance on entire faces gave the weakest impressions of ‘Tired.’ When three images featuring oily and shiny faces were compared, there was a significant difference between images with oily and shiny T‐zones, and oily shine on entire faces in impressions of ‘Tired.’ Thus, images of faces with oily and shiny T‐zones gave a stronger impression of ‘Tired’ than images of faces with oily shine on entire faces (Table 1, Figure 6).

‘Radiant skin’ was felt most strongly when the entire face was radiant, followed by images of faces with radiant cheeks, faces that were oily shine on entire, faces with oily and shiny cheeks and radiant T‐zone, and faces with oily and shiny T‐zones. Matte faces gave the weakest impressions of ‘Radiant skin.’ There was no significant difference in impressions of ‘Radiant skin’ between faces with oily and shiny face cheeks and radiant T‐zones (Table 1, Figure 6).

‘Oily and shiny skin’ was felt most strongly in images of oily shine on entire face skin, followed by faces with oily and shiny cheeks and T‐zones, and the three conditions of radiant faces. Images of matte faces gave the weakest impressions of ‘Oily and shiny skin.’ There was no significant difference between images of faces with oily and shiny cheeks and T‐zones (Table 1, Figure 6).

‘Bright skin’ was felt most strongly in images of faces that were radiance on entire face, followed by faces with radiant T‐zones and those that were oily shine on entire faces, those with radiant cheeks, and images of faces with oily and shiny cheeks and T‐zones. Images of matte faces gave the weakest impressions of ‘Bright skin.’ There was no significant difference between facial images with oily shine on entire faces and those with radiant T‐zones or between faces with oily and shiny T‐zones and those with oily and shiny cheeks (Table 1, Figure 6).

## Discussion

The first aim of the present study was to examine whether the types of facial skin reflection (i.e. radiant, oily and shiny, and matte) influence facial attractiveness and other affective impressions and the second aim was to examine the influence of the position of the reflection on the face. In this study, we performed two psychological experiments where we examined effects of skin reflection types and positions on estimated age. We comprehensively investigated those effects on facial attractiveness and on nine other affective impressions, including the desire to compliment, looking happy, likableness, looking tired, looking feminine, looking healthy, skin radiance, oily shine and skin brightness. We show for the first time that radiance, a specific type of facial reflection, has a positive effect on a wide variety of impressions of the face by empirical data. Furthermore, we revealed that the position of radiance on the face differentially improves impressions of health and femininity. This is important because understanding the association between facial impressions and facial radiance could provide consumer the value of facial radiance achieved by using skincare and base makeup.

### Age estimation

Images with radiance on entire faces were evaluated as the youngest. The other two conditions featuring radiance, radiant cheeks and T‐zones, were rated younger than images of the matte face. By contrast, no significant difference was found between images of the three oily and shiny faces and the matte face. Therefore, these results suggest that not all types of facial gloss give an impression of youth.

With regard to the effect of position of the gloss, no significant difference was found between images of faces with radiant cheeks and T‐zones, or between those of faces with the oily and shiny cheeks and T‐zones. Therefore, the position of gloss had no effect on age estimation.

Nevertheless, there was an additive effect of radiance of the cheeks and the T‐zone. As mentioned above, images of faces with radiant cheeks and T‐zones were rated as younger than those of matte faces. In addition, the faces with radiance on entire facial skin were rated younger than those with radiance either only on cheeks or only on T‐zones. These results suggest that images of radiant cheeks and T‐zones in radiance on entire faces yielded additive effects to help the faces appear younger still.

By contrast, no such additive effect was found for images of oily and shiny faces as no significant difference was found among the three oily and shiny conditions. Hence, the additive effect of reducing estimated age seems to be unique to radiance.

Previous research has suggested that facial information, such as skin brightness, reflection and colour [19, 21], facial contrast [29], skin colour distribution [2], wrinkles around the eyes, spots and skin sag [30], influence estimated age. Further, Nagasaki et al. showed that females in their 40s tend to be evaluated as older as skin sebum increases [30]. Arakawa et al. suggested that skin texture becomes rough with increasing skin sebum [31]. Skin with a higher degree of specular reflection and lower diffuse reflection has rough texture, which gives the impression of oily and shiny gloss [17]. A previous study [19] showed that images of people with facial skin patches with emphasized luminance of specular reflections were estimated to be older than those without specular reflections, and the differences increased in subjects aged 40 years and older (figures 5, 7 and 9 in the relevant study). This suggests that the specular reflection of the skin accentuates the roughness of its texture, which is a feature of middle age that emphasizes the impression of old age. It has been suggested that the impression of a radiant skin requires both types of reflections, specular and diffuse, as in younger people [17]. However, a previous study [19] did not reveal such effects. This seems inconsistent with the results of our experiment. One possible reason for this is the difference in the size of the stimulus images used. We used photos of the entire face, whereas Ref. [19] used images of skin patches, which enhanced the texture and wrinkles of the skin, even in the young people, because it presented to the observer in a magnified manner. As a result, specular reflection might have caused the feeling of oily and shiny skin rather than radiant skin. It was unclear whether images of skin patches with specular and diffuse reflections in Ref. [19] were considered representative of oily and shiny skin. However, this does not contradict the results here, where radiant skin made the female models look younger than oily and shiny skin.

### Impressions of the face

Images of radiant faces gave the participants had the most positive impressions across all questions for images of radiant faces. In addition, oily and shiny faces evoked more positive impressions than matte faces. Therefore, radiance in faces prompts positive impressions in observers.

Why did facial skin radiance enhance positive impressions? One possible reason is from a biological perspective. Radiant skin is produced by combining diffused reflection and specular reflection, and skin with diffused reflection has the feature of decreasing melanin, increasing moisture, and inducing finer texture and good blood circulation [17]. Skin in such a condition is thus interpreted as healthy. Previous studies reported an association between facial attractiveness and health. Some studies have concluded that facial symmetry and averageness contribute to facial attractiveness [5, 6]. These features are interpreted as cues expressing genetic heterozygosity, which can signal an outbreed mate or provide genetic diversity in defence against parasites [6]. Other studies suggest a relation between visible skin features, attractiveness and impressions of healthiness. A previous study indicates that attractiveness increases with fineness of the texture of the skin and skin chroma [1]. The authors of another study proposed that skin texture is a cue for fertility and health. It has also been reported that skin blemishes diminish impressions of healthiness and attractiveness [7]. Healthy individuals thus have higher value as mates and thus are considered attractive. These studies support the claim here that faces with radiant skin improve positive impressions in observers.

The questionnaires on evaluating impressions contained sensory words, such as ‘Radiant skin’, ‘Oily and shiny skin’ and ‘Bright skin’. The impression of ‘Oily and shiny skin’ was felt more strongly towards faces with shiny gloss. This tendency indicates that the method of creating stimulus images of oily and shiny faces in previous studies can appropriately evoke this impression. The impression of a ‘Radiant skin’ was felt most strongly in faces that had radiance on entire face, followed by faces with radiant cheeks. However, faces with oily and shiny cheeks, and oily shine on entire faces provided the same, or stronger, feeling of ‘Radiant skin’ than faces with radiant T‐zones, even though the two image conditions featured oily and shiny faces. The impression of ‘Radiant skin’ was felt more strongly in faces with radiant cheeks than those with radiant T‐zones. This suggests that the type of gloss as well as its position affects the impression of ‘Radiant skin.’ The impression of a ‘Bright skin’ was felt more strongly when gloss was in a larger area and faces with radiant T‐zones and oily shine on entire faces evoked the same degree of impression. Therefore, both gloss type and area can influence the impression of ‘Bright skin.’ In addition, a strong impression of ‘Bright skin’ did not always accompany that of ‘Radiant skin’ to participants.

The results for the three types of faces with radiance showed that facial images with radiance on entire faces evoked the most positive impressions, whereas there was nearly no difference between impressions of faces with radiant cheeks and those with radiant T‐zones. A synergetic effect, combining radiance on the cheeks with that radiance on the T‐zone, evoked a stronger impression among the participants, as shown in all abstract and sensory words used to describe their feelings for images in these categories, except for the ‘Oily and shiny skin’ impression.

The responses to some questionnaires showed differences in degree between images of faces with radiant cheeks and T‐zones. Impressions of ‘Radiant skin’ and ‘Healthy’ were felt more strongly in the former than the latter (Figure 6, Table 1). On the contrary, impressions of ‘Feminine’ and ‘Bright skin’ were felt more strongly in faces with radiant T‐zones face than those with radiant cheeks. These results indicate that radiance could evoke a stronger positive impression in the observer when evident on both cheeks and the T‐zone, and different positions of radiance can enhance different impressions.

Why did different positions of skin radiance improve different positive impressions of ‘Feminine’ and ‘Healthy’? A previous study [32] provides a suggestion. The authors showed the results of a principal axis factor analysis of the rated facial traits. In the process of the analysis, questionnaire items of ‘attractiveness’, ‘healthiness’ and ‘smiles’ were contained in the same factor but ‘sexual dimorphism’ was contained in another factor. The implication of these results supports the possibility that different positions of radiance in the T‐zone and the cheeks served as cues to evoke impressions of ‘Feminine’ and ‘Healthy.’

The three types of oily and shiny faces were consistently evaluated more negatively than faces with radiance. Furthermore, there were differences among the conditions of images of oily and shiny faces. Images of faces with oily and shiny T‐zones did not yield more positive impressions among the participants than the other two types of oily and shiny faces. The participants tended to evaluate facial images with oily shine on entire faces more positively than faces with only oily and shiny T‐zones in terms of both sensory expressions and abstract words. The responses to questionnaires on sensory expressions indicate that facial images with oily shine on entire faces evoked stronger impressions in the participants than faces with oily and shiny cheeks. Moreover, facial images oily shine on entire faces evoked the same strength of impression as oily and shiny cheeks in terms of abstract words, and conveyed a less ‘Likable’ impression. These results suggest the effect of the position of oily and shiny gloss on impressions of the face because faces of oily and shiny T‐zones evoked more negative impressions than them of oily and shiny cheek. There was no synergy such that combining oily and shiny gloss on the cheeks and T‐zone increased positive or negative impressions.

Why did faces with oily and shiny skin yield more negative impressions than the faces with radiance? One possible reason is that faces with oily and shiny skin gave a less healthy impression because such skin features low diffused reflection and high specular reflection induced by rough texture, less moisture, and poor blood circulation [17]. In addition, oily and shiny skin is a cause of concern for females when the foundation wears off [20]. In this study, images of entire oily and shiny faces provided weak impression of ‘Likable’ than those with oily and shiny cheeks. This result may be interpreted as oily and shiny gloss on the T‐zone inducing a negative effect. Torizuka et al. claimed that females are concerned about deteriorating foundation specifically on the T‐zone because of the secretion of sebum [20]. This implies that personal experience influenced the link between images of oily and shiny faces, and negative impressions. To examine this suggestion, another experiment is needed involving male observers and female models.

### Cultural effects

One limitation of our present study is that we recruited only Japanese females as models and observers. This limitation leaves the question of cultural effects on how skin reflection influences facial impressions, which has been implied by previous research. For example, a large trial in China has identified self‐evaluated factors relevant to the prevalence of oily skin, which may be specific to the Chinese female population Nouveau‐Richard, [26]. A cross‐cultural survey in cosmetics has reported that French female university students tend to prefer suntanned skin, whereas Japanese ones show the opposite preference Ishimaru [33]. These studies thus may indicate possible cultural differences in the effects we found of skin radiance on facial attractiveness. Therefore, caution should be used before considering the present results as a universal, cross‐cultural tendency. Further clarification of such possible cultural effects may have scientific and industrial significance and is subject to future work.

## Conclusion

This study is the first to empirically show that radiance, a specific type of facial reflection, has a positive effect on a wide variety of impressions of the face. Furthermore, we revealed that the position of radiance on the face differentially improves impressions of health and femininity. Previous studies have suggested that the shape [1, 2, 3, 4], colour [8, 9] and texture [7] of the face are essential factors in forming affective impressions. We have shown here that facial reflection serves as another important factor. Our empirical findings support the enhancement of subjective impressions of faces by improving radiance of a face, which may provide a better understanding of skincare and base makeup.

We hope that a new perspective on the value of facial radiance can be provided by the present scientific evidence on the effects of facial radiance on facial attractiveness and other affective impressions. Consequently, understanding the link between facial impressions and facial radiance could help determine the value of facial radiance achieved by using skincare and base makeup.
